# Supplementary material for: Deformation and seismicity decline before the 2021 Fagradalsfjall eruption
Source: Nature. 2022 Sep 14;609(7927):523–8. doi: 10.1038/s41586-022-05083-4 (PMC9477732; doi:10.1038/s41586-022-05083-4)
Supplement: Supplementary file 1 — The table lists 65 earthquakes on the Reykjanes Peninsula recorded during February to April 2021 of moment magnitude (MW) larger than or equal to 4.0. [file 41586_2022_5083_MOESM1_ESM.docx]

**Sigmundsson et al.:**

**Deformation and seismicity decline before the 2021 Fagradalsfjall eruption**

**Supplementary Data Table 1**

The 65 earthquakes on Reykjanes Peninsula recorded during February – April 2021 of Moment Magnitude, M_W_ ≥ 4.0 (see Methods for description of procedure for estimating the moment magnitudes and the corresponding seismic moments, M_0_). Locations are routine reviewed SIL locations with accuracies generally of 0.002° in latitude, 0.003° in longitude and 0.4 km in depth.

*Magnitudes determined by the US Geological Survey.

| Date | Origin time | Latitude | Longitude | Depth | M_w_ | M_0_ |
| --- | --- | --- | --- | --- | --- | --- |
| 20210224 | 100557.07 | 63.906 | -22.212 | 2.8 | **5.64** | 3.236E+17 |
| 20210224 | 101753.01 | 63.897 | -22.319 | 4.7 | 4.8* | 1.778E+16 |
| 20210224 | 102759.65 | 63.916 | -22.055 | 4.2 | 4.63 | 9.886E+15 |
| 20210224 | 103046.08 | 63.913 | -22.102 | 2.4 | 4.8* | 1.778E+16 |
| 20210224 | 104236.29 | 63.925 | -21.989 | 3.0 | 4.40 | 4.467E+15 |
| 20210224 | 104859.94 | 63.885 | -22.389 | 5.1 | 4.75 | 1.496E+16 |
| 20210224 | 105234.60 | 63.866 | -22.393 | 5.3 | 4.2* | 2.239E+15 |
| 20210224 | 113838.84 | 63.910 | -22.032 | 1.3 | 4.03 | 1.245E+15 |
| 20210224 | 123730.22 | 63.925 | -21.987 | 2.5 | 4.48 | 5.888E+15 |
| 20210226 | 120649.77 | 63.913 | -22.243 | 4.4 | 4.52 | 6.761E+15 |
| 20210226 | 122413.25 | 63.910 | -22.242 | 6.4 | 4.30 | 3.162E+15 |
| 20210226 | 135154.69 | 63.919 | -22.225 | 6.2 | 4.02 | 1.202E+15 |
| 20210226 | 151030.59 | 63.929 | -22.218 | 6.0 | 4.11 | 1.641E+15 |
| 20210226 | 164830.64 | 63.908 | -22.238 | 6.6 | 4.34 | 3.631E+15 |
| 20210226 | 165628.87 | 63.905 | -22.272 | 5.7 | 4.05 | 1.334E+15 |
| 20210226 | 200809.64 | 63.923 | -22.221 | 5.7 | 4.35 | 3.758E+15 |
| 20210226 | 223843.64 | 63.925 | -22.219 | 4.5 | 4.88 | 2.344E+16 |
| 20210227 | 41410.24 | 63.925 | -22.209 | 5.0 | 4.12 | 1.698E+15 |
| 20210227 | 80744.43 | 63.912 | -22.236 | 5.7 | **5.15** | 5.957E+16 |
| 20210227 | 81850.36 | 63.890 | -22.294 | 3.9 | 3.99 | 1.084E+15 |
| 20210228 | 1914.99 | 63.920 | -22.233 | 5.4 | 4.73 | 1.396E+16 |
| 20210228 | 75436.44 | 63.913 | -22.228 | 5.2 | 4.00 | 1.122E+15 |
| 20210228 | 113227.99 | 63.922 | -22.211 | 5.2 | 4.29 | 3.055E+15 |
| 20210228 | 153931.24 | 63.935 | -22.199 | 8.2 | 4.04 | 1.288E+15 |
| 20210228 | 162956.75 | 63.942 | -22.186 | 4.1 | 4.17 | 2.018E+15 |
| 20210228 | 190137.19 | 63.924 | -22.188 | 5.0 | 4.68 | 1.175E+16 |
| 20210301 | 13137.71 | 63.918 | -22.209 | 4.8 | 4.88 | 2.344E+16 |
| 20210301 | 121217.32 | 63.917 | -22.214 | 5.3 | 4.02 | 1.202E+15 |
| 20210301 | 141237.48 | 63.930 | -22.198 | 5.6 | 4.17 | 2.018E+15 |
| 20210301 | 163546.12 | 63.941 | -22.155 | 3.2 | **5.02** | 3.802E+16 |
| 20210302 | 25322.84 | 63.934 | -22.191 | 3.0 | 4.26 | 2.754E+15 |
| 20210302 | 30532.58 | 63.914 | -22.236 | 6.3 | 4.68 | 1.175E+16 |
| 20210302 | 53611.78 | 63.922 | -22.223 | 6.0 | 4.31 | 3.273E+15 |
| 20210302 | 61556.99 | 63.924 | -22.210 | 5.2 | 4.03 | 1.245E+15 |
| 20210302 | 101245.22 | 63.929 | -22.178 | 5.3 | 4.04 | 1.288E+15 |
| 20210303 | 21201.89 | 63.911 | -22.229 | 5.4 | 4.14 | 1.820E+15 |
| 20210304 | 5908.17 | 63.900 | -22.253 | 6.4 | 3.97 | 1.012E+15 |
| 20210304 | 53617.91 | 63.898 | -22.309 | 5.7 | 4.18 | 2.089E+15 |
| 20210304 | 85407.22 | 63.891 | -22.257 | 6.7 | 4.64 | 1.023E+16 |
| 20210304 | 191422.43 | 63.857 | -22.437 | 5.2 | 4.13 | 1.758E+15 |
| 20210305 | 115051.82 | 63.883 | -22.326 | 4.1 | 4.08 | 1.479E+15 |
| 20210307 | 4239.41 | 63.898 | -22.255 | 6.6 | 4.06 | 1.380E+15 |
| 20210307 | 14006.66 | 63.894 | -22.289 | 6.2 | 4.20 | 2.239E+15 |
| 20210307 | 20126.40 | 63.891 | -22.333 | 6.4 | **5.17** | 6.383E+16 |
| 20210307 | 23336.75 | 63.896 | -22.274 | 6.5 | 4.04 | 1.288E+15 |
| 20210307 | 24041.59 | 63.878 | -22.370 | 4.9 | 4.02 | 1.202E+15 |
| 20210307 | 24534.08 | 63.887 | -22.366 | 4.8 | 4.37 | 4.027E+15 |
| 20210307 | 25043.73 | 63.873 | -22.425 | 2.3 | 4.48 | 5.888E+15 |
| 20210307 | 25431.72 | 63.879 | -22.446 | 4.6 | 4.3* | 3.162E+15 |
| 20210307 | 170611.26 | 63.911 | -22.242 | 5.1 | 4.23 | 2.483E+15 |
| 20210309 | 230143.82 | 63.886 | -22.259 | 6.4 | 4.0* | 1.122E+15 |
| 20210310 | 31438.60 | 63.884 | -22.295 | 5.7 | **5.19** | 6.839E+16 |
| 20210310 | 85000.76 | 63.889 | -22.260 | 4.0 | 4.49 | 6.095E+15 |
| 20210310 | 150026.82 | 63.883 | -22.254 | 4.6 | 4.20 | 2.239E+15 |
| 20210311 | 85315.57 | 63.842 | -22.571 | 4.7 | 4.60 | 8.913E+15 |
| 20210312 | 74301.98 | 63.878 | -22.294 | 3.7 | 4.58 | 8.318E+15 |
| 20210312 | 223950.87 | 63.874 | -22.281 | 6.4 | 4.19 | 2.163E+15 |
| 20210313 | 13432.93 | 63.867 | -22.286 | 5.7 | 4.46 | 5.495E+15 |
| 20210313 | 220620.34 | 63.882 | -22.267 | 4.2 | 4.04 | 1.288E+15 |
| 20210314 | 44048.89 | 63.877 | -22.284 | 4.2 | 4.16 | 1.950E+15 |
| 20210314 | 123436.30 | 63.866 | -22.285 | 5.0 | 4.63 | 9.886E+15 |
| 20210314 | 141525.05 | 63.863 | -22.338 | 3.3 | **5.33** | 1.109E+17 |
| 20210314 | 143822.86 | 63.883 | -22.457 | 3.8 | 4.09 | 1.531E+15 |
| 20210315 | 223154.94 | 63.911 | -22.244 | 4.8 | 4.20 | 2.239E+15 |
| 20210420 | 230553.77 | 63.877 | -22.388 | 5.7 | 4.00 | 1.122E+15 |
